# Active compounds of herbs ameliorate impaired cognition in APP/PS1 mouse model of Alzheimer’s disease

**DOI:** 10.18632/aging.102522

**Published:** 2019-12-09

**Authors:** WenJun Yu, ShengJun An, TieMei Shao, HongJun Xu, HongXu Chen, JunDa Ning, YongJie Zhou, XiQing Chai

**Affiliations:** 1 Department of Neurology, The First Hospital of Hebei Medical University, Shijiazhuang, Hebei, China; 2 Research Center, Hebei University of Chinese Medicine, Shijiazhuang, Hebei,China; 3 Hebei Plant Bioreactor Preparation Technology Engineering Center, Shijiazhuang, Hebei, China; 4 Hebei Chemical and Pharmaceutical College, Shijiazhuang, Hebei, China

**Keywords:** Alzheimer’s disease, iron metabolism-related proteins, Chinese herb active compounds

## Abstract

Alzheimer’s disease (AD), the most common cause of dementia, is a neurodegenerative disorder characterized by amyloid plaque accumulations, intracellular tangles and neuronal loss in certain brain regions. It has been shown that a disturbance of normal iron metabolism contributes to the pathophysiology of AD. However, the mechanism underlying abnormal iron load in the brain of AD patients is unclear. The frontal cortex, an important brain structure for executive function, is one of the regions affected by AD. We investigated the beneficial effects of active compounds of Epimedium, Astragaoside and Puerarin on iron metabolism in the frontal cortex of six-month-old APPswe/PS1_ΔE9_ (APP/PS1) double transgenic mouse, a model of AD. Treatment with the active compounds reduced cognitive and memory deficits and damaged cell ultrastructure in APP/PS1 mice. These beneficial effects were associated with changes in expression levels of iron metabolism proteins in the frontal cortex, including divalent metal transporter with iron response element (DMT1-with IRE), divalent metal transporter without iron response element (DMT1-without IRE), transferrin (TF) and transferring receptor 1 (TfR1); three release proteins including the exporter ferroportin 1 (Fpn1), ceruloplasmin (CP) and hephaestin (HEPH), one increased storage iron protein ferritin and one iron regulating hormone hepcidin. These findings suggest that the active compounds improve cognition and memory in brain neurodegenerative disorders and these beneficial effects are associated with reduced impairment of iron metabolism. This study may provide a new strategy for developing novel drugs to treat AD.

## INTRODUCTION

AD is a devastating neurodegenerative disorder that leads to cognitive impairment and dementia. It is established that the pathological hallmarks of AD are the deposition of β-amyloid (Aβ) in senile plaques (SPs) and the appearance of neurofibrillary tangles (NFTs) produced by hyperphosphorylated tau protein in selective brain regions. However, due to the complexity of the molecular pathogenesis of AD and multiple factor interactions, the mechanisms underlying the etiology and development of AD is not fully elucidated. Thus, the effective therapies for AD are to be developed.

The etiology of AD involves a variety of pathophysiological factors, among which dysfunctional homeostasis of transition metals, particularly altered iron metabolism, is one of the causes that play an important role in the pathogenesis of AD [1, 2]. Iron is a redox active metal that exists in the ferrous or ferric state, which is involved in many metabolic functions [3]. Recently, it has been shown that abnormally increased iron contents are observed in the brain of patients suffering from AD [4, 5]. A meta-analysis of 20 magnetic resonance imaging (MRI) studies further support a relationship between high iron content in the central nervous system and the pathogenesis of AD [6]. Also, age-related increase in iron levels in the brain has been proposed as a biomarker of cognitive defect and progression of neuroanatomical aging in healthy adults [6]. Previous studies have demonstrated that redox-active iron is closely associated with AD plaques and neurofibrillary tangles [4, 7]. In addition, iron accumulation is associated with microgliosis and correlated with increased damage to hippocampal neurons in CA1 region [8]. Therefore, it is likely that abnormal iron metabolism associated with AD is affected by the altered expression of iron metabolism-associated proteins. The disturbances of iron metabolism might occur at several biological pathways, including iron uptake, release, storage and regulation of functional iron proteins at both the cellular and systemic levels [9].

Deferoxamine (DFO) is an iron chelator that significantly alleviates the symptoms of patients with AD [10, 11]. However, DFO is not suitable for oral intake due to its side effects. Chinese herbal medicine compatibility is the predominant form for clinical medication for three thousand years. Epimedium, Astragalus, Puerarin are highly valued traditional Chinese medicines and have been widely used in clinics because of their neuroregulatory and neuroprotective effects. For example, Epimedium may reduce Aβ-induced neurotoxicity through attenuation of Aβ deposition in amyloid plaques in the cortex and the hippocampus of transgenic mice [12–15]. *Astragalus membranaceus* inhibits oxidative stress induced by metal [16] and antagonizes Aβ1-42 neurotoxicity in Human SK-N-SH neuroblastoma cells [17]. Puerarin, an isoflavone purified from the root of *Pueraria lobata*, has been reported to attenuate learning and memory impairments in the transgenic mouse model of AD [18] and could protect neurons from oxidative stress-induced apoptosis [19]. Collectively, these compounds have the potential to impact the development and progression of AD.

This study aimed to investigate the roles of active compounds, Epimedium, Astragalus and Puerarin in modulating iron metabolism-related proteins in APP/PS1 double transgenic mouse mice, a model of AD. The APP/PS1 double transgenic mice expresses 2 major mutations in the human APP, as well as 2 human PS1 mutations knocked-in into the mouse PS1 gene in a homozygous manner. These mice were bred in a C57BL/6J (C57) background. By using the Morris water maze test (MWM) and novel object recognition (NOR) test, we determined whether the active compounds treatment attenuate the cognitive and memory deficits in this AD mouse model. Following the behavioral tests, the Aβ-42 accumulation and ultrastructure in the mice frontal cortex were determined. Furthermore, to clarify the molecular mechanisms underlying abnormal iron levels associated with AD in the brain, the expression levels of iron metabolism proteins including 4 major iron uptake proteins, 3 release proteins, one storage iron protein and one iron regulating hormone were compared between animals with or without administration of the active compounds of Epimedium, Astragalus and Puerarin.

## RESULTS

### Behavioral examinations

**Figure 1:**
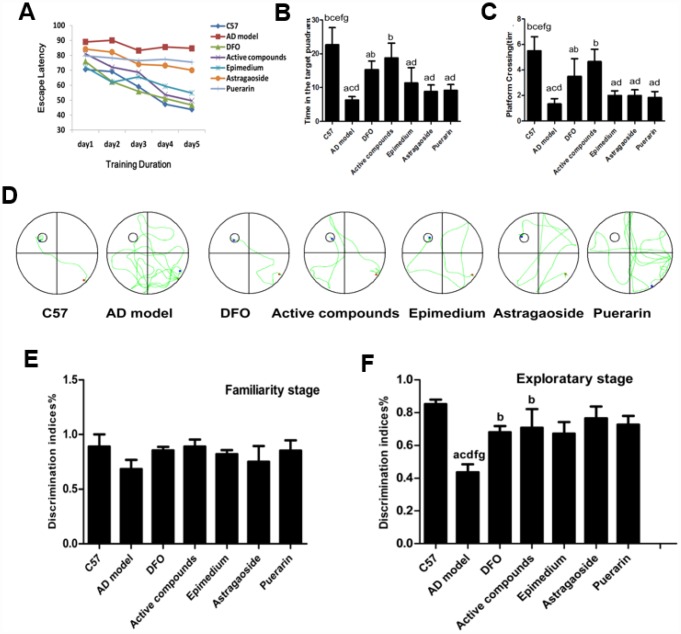
**Behavioral performance of animals in the Morris water maze.** (**A**) Average escape latencies. (**B**) Percentage time spent in the quadrant that previously contained the platform. (**C**) Number of crossings to the previous location of the platform. (**D**) Representative swimming paths on day 5 of the place navigation trial were recorded with a video tracking system. (**E**) familiarity stage (**F**) exploratory stage. (n=6 per group). Data are presented as the mean± standard error (n=6 per group). (**A**) P < 0.05, vs. C57; (**B**) P < 0.05, vs. AD model; (**C**) P < 0.05, vs. DFO; (**D**) P < 0.05, vs. Active compounds; (**E**) P < 0.05, vs. Epimedium; (**F**) P < 0.05, vs. Astragaoside, (**G**) P < 0.05, vs. Puerarin. DI = TN/(TN + TF), TN as novel object and TF as familiar objects. discrimination index (DI).

The Morris water maze test was conducted firstly to investigate whether treatment with these active compounds of Epimedium, Astragaoside, Puerarin or combination of these three herbs reduced cognitive deficits in AD transgenic model mice. The analysis of the place navigation trial showing escape latencies was conducted from day 1 to day 5 in all groups (Figure 1A). The results showed that the AD transgenic model mice exhibited longer escape latencies than the C57 wild-type group (P < 0.05). Treatment with the active compounds or DFO significantly reduced escape latencies in the AD transgenic mice (P < 0.05). These results suggest that the active compounds treatment ameliorate the impairment of spatial learning and memory in the AD transgenic model mice.

The analysis of the spatial probe trial reveals that the time spent in the target quadrant (Figure 1B) and platform-crossing times in the target quadrant (Figure 1C). The AD transgenic mice spent significantly less time in the quadrant containing the platform than the C57 group, active compounds and DFO group (P<0.05). Furthermore, the number of crossings over the previous location of the platform was reduced in the AD model group compared with other groups (P<0.05) while the escape latencies between the active compounds and DFO groups did not differ significantly. These results also showed that the mice treated with the active compounds or DFO spent more time in the previous quadrant than in each unilateral group. Figure 1D displayed the representative swimming paths of mice in each group, indicating that the AD transgenic model mice performed more unnecessary swimming. The platform crossing and the time spent in the target quadrant of mice administered with active compounds were much higher than the three unilateral groups. All these results indicate that the ability of the mice to apply spatial cues to the localization of the platform was impaired in the AD group (P<0.05) and the cognition was improved by treatment with active compounds of Epimedium, Astragaoside, and Puerarin and DFO. There was no significant difference between the active compounds and DFO groups.

To determine if active compounds of Epimedium, Astragaoside, Puerarin ameliorate episodic memory decline in AD model mice, during the familiarity stage of novel object recognition (NOR), the discrimination indices showed no difference in time spent between groups when two identical objects were explored and indicated a significant effect in the exploratory stage (P<0.05) for exploration of a new object. Whereas, the episodic memory impairment was evident in the AD transgenic mice by showing lower discrimination index (Figure 1E and 1F). This impaired episodic memory in the AD transgenic model group was improved by treatment with the active compounds and DFO. There was no significant difference between the active compounds and DFO groups in the discrimination indices.

### Neuroprotective effects of the active compounds of Epimedium, Astragaoside and Puerarin in the cortex of AD transgenic model mice

**Figure 2:**
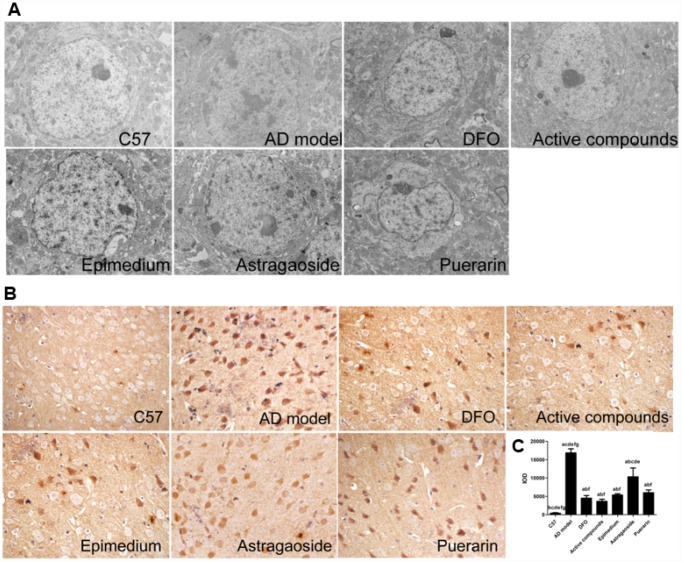
**Active compounds treatment reduces ultrastructural impairment and decreases Aβ-42 expression in the AD model mouse frontal cortex.** (**A**) The C57 group neural cell was normal group as exhibited by intact membranes, uniform cytoplasm, and complete organelle structure. The AD group was showing that the nuclear shrinkage and deformed, the mitochondrial cristae fused with partial membranes, rough endoplasmic reticulum degranulated. The active compounds and DFO group were similar as C57 group. The ultrastructure of Epimedium, Astragaoside, and Puerarin group were similar, presenting partial neural edema or loss normal morphology, the nuclear shrinkage and deformed slightly(magnification:4000×). (**B**) The IOD value showing that the active compounds treatment reduces Aβ-42 expression in the AD model mouse frontal cortex. No significantly different between DFO and active compounds group. All data are expressed as the mean ± SD. Intergroup differences were compared with multivariate analysis of variance followed by the least significant difference test. (**A**) P < 0.05, vs. C57; (**B**) P < 0.05, vs. AD model; (**C**) P < 0.05, vs. DFO; (**D**) P < 0.05, vs. Active compounds; (**E**) P < 0.05, vs. Epimedium; (**F**) P < 0.05, vs. Astragaoside, P < 0.05, vs. Puerarin.

TEM was used to observe the ultrastructure in frontal cortex (Figure 2A). In C57 mice, the neuronal ultrastructure was normal as indicated by intact membranes, uniform cytoplasm, and complete organelle structure. In the AD transgenic mice, the nucleus shrunk and deformed, the mitochondrial cristae fused with partial membranes, the rough endoplasmic reticulum degranulated, mitochondria swelled, membrane ridges disappeared and cytoplasm vacuolization were observed, all of which indicates the damage of ultrastructure in neurons. The neuron ultrastructure of the Epimedium, Astragaoside, and Puerarin treatment groups were similar, presenting partial neural edema or loss of normal morphology. Additionally, the nucleus shrunk and deformed slightly, the mitochondria occasionally swelled and deformed or the endoplasmic reticulum were slightly dilated. There were no significant differences between the three groups. The ultrastructure of active compounds and DFO group were similar as C57 mice, showing relatively normal karyomorphism and membrane structure. Additionally, the organelles are relatively complete in morphology. The result suggests that the ultrastructure of frontal cortex in AD transgenic mice was obviously damaged and the compounds of active Epimedium, Astragaoside, and Puerarin reduced these damaged ultrastructures in frontal cortex.

Immunohistochemical staining revealed that the Aβ-42 expression was higher in the cerebral frontal cortex of the AD transgenic model group compared with the C57 wild-type group (Figure 2B and 2C). Treatment with either the active compounds, Epimedium, Astragaoside, Puerarin or DFO significantly reduced Aβ-42 expression (P<0.05). The active compounds resulted in a further decrease in the expression of Aβ-42, which was similar to that in the DFO group.

### The effects of the active compounds of Epimedium, Astragaoside and Puerarin on the expression of iron metabolism related proteins

**Figure 3:**
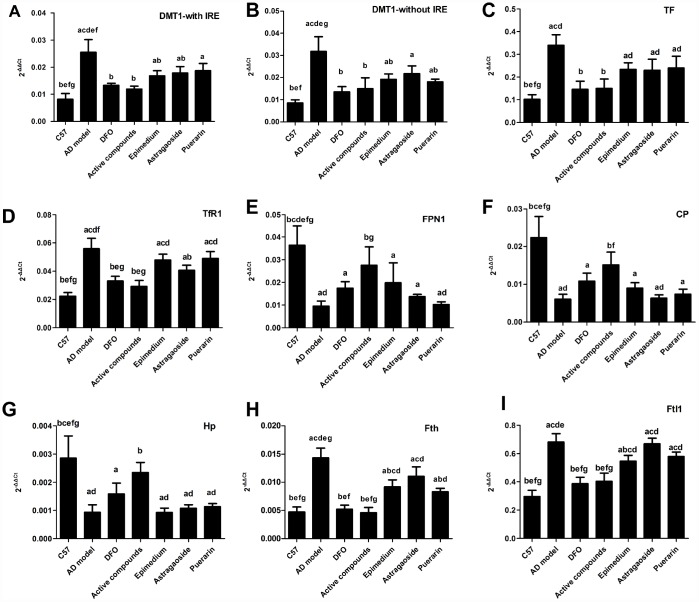
**Effects of the active compounds containing the effective components of Epimedium, Astragaoside and Puerarin on the mRNA expression levels of the iron uptake protein including DMT1-with IRE, DMT1-without IRE, TF, TfR1, iron release protein including FPN1, CP, HEPH; iron storage protein Ferritin, including Fth and Ftl1 in the cerebral cortex of AD transgenic mice.** All data are expressed as the mean ± SD. Inter group differences were compared with multivariate analysis of variance followed by the least significant difference test. (**A**) P < 0.05, vs. C57; (**B**) P < 0.05, vs. AD model; (**C**) P < 0.05, vs. DFO; (**D**) P < 0.05, vs. Active compounds; (**E**) P < 0.05, vs. Epimedium; (**F**) P < 0.05, vs. Astragaoside, P < 0.05, vs. Puerarin.

Figure 3 shows Real time-PCR analysis of the mRNA expression of essential iron uptake protein DMT1-with IRE, DMT1-without IRE, TF, TfR1, essential iron release protein FPN1, CP, HEPH and the storage protein ferritin. The mRNA expression levels of these 4 iron uptake protein in the AD model group were higher compared with the C57 group, which was reversed by treatment with either DFO or active compounds (P<0.05). Moreover, the effects of the DFO or active compounds were powerful than either of Epimedium or Astragaoside. And the mRNA expression of the Epimedium, Astragaoside groups were also lower than the AD model groups (Figure 3A, P<0.05). The DMT1-without IRE result showed that the AD model group were lower than the DFO and active compounds groups. The Epimedium or Puerarin group were lower than the AD model group (Figure 3B, P<0.05). The TF mRNA expression showed that the DFO and active compounds groups were lower than the AD model group. The mRNA expression of three unilateral groups were higher than the active compounds (Figure 3C, P<0.05). The mRNA expression of TFR1 showed that DFO and active compounds groups were lower than the AD model group and the Astragaoside group were also lower than the AD model group (Figure 3D P<0.05). Among the three main release proteins, the FPN1 mRNA expression in the DFO and active compounds groups were lower compared with those in AD model group (Figure 3E P<0.05). The CP mRNA expression showed that AD model group was lower than the DFO and active compounds groups (Figure 3F, P<0.05). The HEPH mRNA expression result showed that the active compounds were higher than the AD model group and the three unilateral groups (Figure 3H, P<0.05). The mRNA expression of storage iron protein ferritin included Ftl1 and Fth. And the results showed that the mRNA expression in the AD model group was higher than the DFO and active compounds groups and three unilateral groups were higher than the active compounds. There was no significant difference between the active compounds and DFO groups. However, in terms of the mRNA expression of FPN1, CP and HEPH the DFO group was lower than the C57 group.

**Figure 4:**
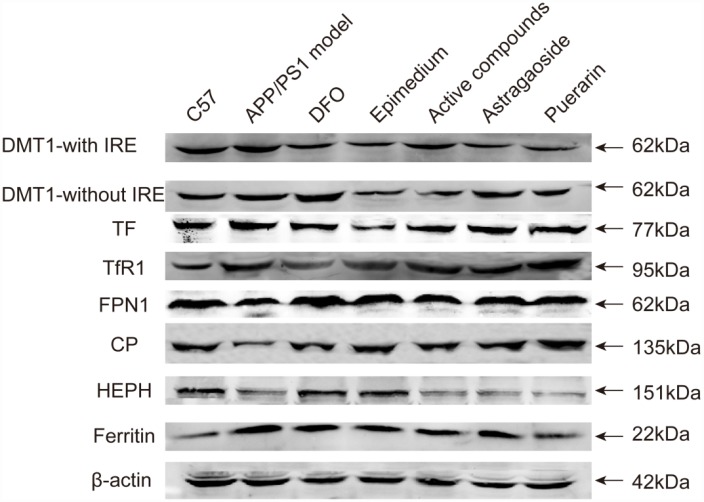
**The protein relative expression levels of the iron uptake protein DMT1-with IRE, DMT1-without IRE, TF, TfR1, iron release protein FPN1, CP, HEPH; iron storage protein Ferritin; in the cerebral cortex of AD transgenic mice after the active compounds containing the effective components of Epimedium, Astragaoside and Puerarin.**

Western blot analysis indicated that in the AD model group that the protein relative levels of iron uptake proteins DMT1-with IRE, DMT1-without IRE, TF and TFR1 were higher than the C57 control group (Figure 4). Most of enhanced protein expressions including DMT1-with IRE, DMT1-without IRE and TF were significantly reduced by the active compounds group. Among the iron release protein FPN1, CP, HEPH, the expressions of these proteins in the AD model group were lower than the C57 group. Whereas, the protein relative levels in the active compounds were higher than AD transgenic model group. Likewise, the storage protein ferritin in the active compounds was lower in the C57 group than the AD model group. There was no significant difference between the active compounds and DFO groups in the protein relative levels. Western blot assay results were consistent with the results of Real-time PCR except for TFR1.

### Effects of the active compounds of Epimedium, Astragaoside and Puerarin on spatial distribution of iron metabolism-related proteins in the frontal cortex

**Figure 5:**
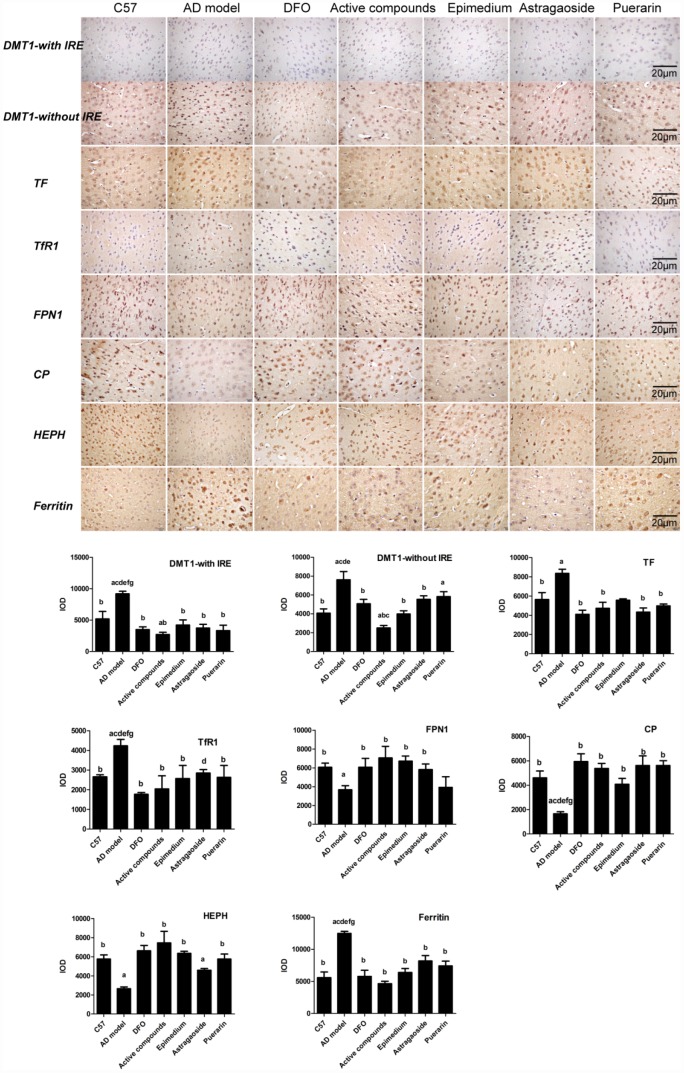
**Effects of the active compounds containing the effective components of Epimedium, Astragaoside and Puerarin on the morphological distribution of the iron uptake protein DMT1-with IRE, DMT1-without IRE, TF, TfR1, iron release protein FPN1, CP, HEPH; iron storage protein Ferritin in the cerebral cortex of AD transgenic mice.** All data are expressed as the mean ± SD. Intergroup differences were compared with multivariate analysis of variance followed by the least significant difference test. (**A**) P < 0.05, vs. C57; (**B**) P < 0.05, vs. AD model; (**C**) P < 0.05, vs. DFO; (**D**) P < 0.05, vs. Active compounds; (**E**) P < 0.05, vs. Epimedium; (**F**) P < 0.05, vs. Astragaoside, P < 0.05, vs. Puerarin. (immunohistochemical staining, magnification 200×).

Immunohistochemical staining revealed that the Integral optical density (IOD) of DMT1-with/without IRE, TF, ferritin, hepcidin expression in AD transgenic model group were higher than in C57 wild-type group (Figure 5, P < 0.05), while the IOD of FPN1, HEPH, CP were lower (P < 0.05) in the AD transgenic model group. These enhanced expressions were reversed by either Epimedium, Astragalus, Puerariae, active compounds or DFO in the AD transgenic model group (P < 0.05). FPN1, HEPH, CP expression was higher in the Epimedium, Astragalus, Puerariae, active compounds and DFO groups than in the AD transgenic model group (P < 0.05). DMT1-with/without IRE, TF, ferritin, hepcidin expression was higher (P < 0.05), but FPN1, HEPH, CP expression was lower (P < 0.05) in the Epimedium, Astragaoside, and Puerarin groups compared with the DFO group and active compounds. There was no significant difference of IOD in each group about TfR1. No significant difference was detected in DMT1-with/without IRE, TF, ferritin, hepcidin and FPN1 expression in AD transgenic model mice frontal cortex between DFO and active compounds groups (P > 0.05).

### The expression of iron metabolism related hormone hepcidin

**Figure 6:**
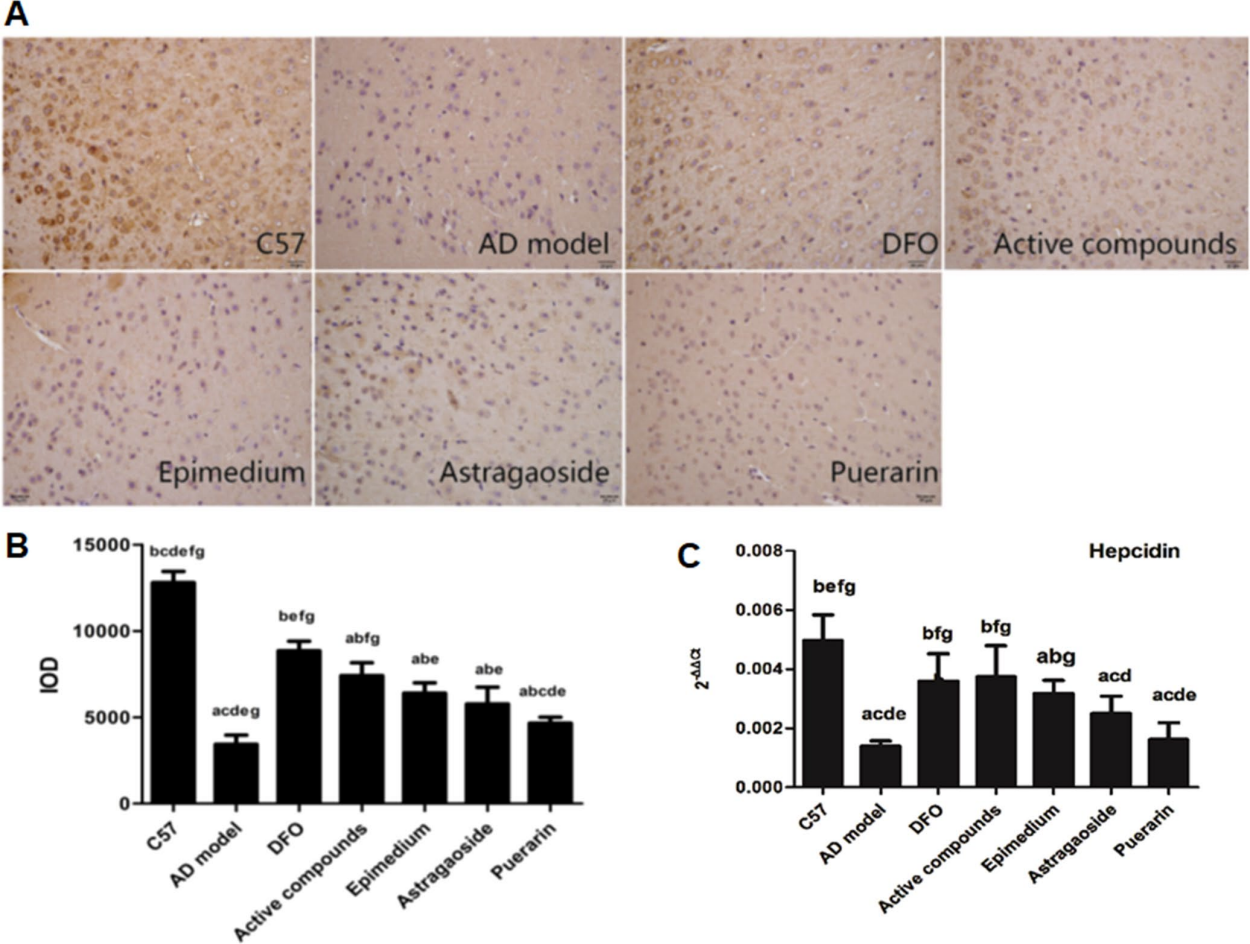
**Effects of the active compounds containing the effective components of Epimedium, Astragaoside and Puerarin on the protein expression levels of the iron regulated hormone hepcidin in the cerebral cortex of AD transgenic mice using the immnohistochemical staining and RT-PCR.** (**A** and **B**) immunostaining images and IOD values of hepcidin. (**C**) mRNA expression levels of the iron regulated hormone hepcidin AD transgenic mice. All data are expressed as the mean ± SD. Intergroup differences were compared with multivariate analysis of variance followed by the least significant difference test. (**A**) P < 0.05, vs. C57; (**B**) P < 0.05, vs. AD model; (**C**) P < 0.05, vs. DFO; (**D**) P < 0.05, vs. Active compounds; (**E**) P < 0.05, vs. Epimedium; (**F**) P < 0.05, vs. Astragaoside, P < 0.05, vs. Puerarin. (immunohistochemical staining, magnification × 200).

The immunohistochemical analysis of Hepcidin revealed that it was reduced in the AD model group compared with the C57 group, and DFO and active compounds groups displayed increased levels of IOD when compared with the AD model group (Figure 6A and 6B, P<0.05).

The mRNA expression of hepcidin has been proposed to have a central role in the homeostasis of iron levels in the brain by regulating the expression of proteins that are related to iron levels in the brain [20, 21]. Real-time PCR analysis of hepcidin showed that the mRNA expression was higher in the C57 group when compared to the AD model group, (Figure 6C, P<0.05). In contrast, the mRNA expression of DFO and active compounds groups were higher than the AD model group (P<0.05). The mRNA expression of Epimedium, Astragaoside, Puerarin group were lower than the C57 group and the Astragaoside and Puerarin groups were higher than the active compounds groups.

## DISCUSSION

The major findings of the present study are as follows: 1) there was significant impairment in learning, spatial memory and episodic memory in the AD model mice. Three months of the active compounds treatment significantly improved these adverse effects; 2) Behavior impairment was accompanied by ultrastructural damages and increased Aβ-42 deposition in the cerebral prefrontal cortex, which also could be ameliorated by treatment with active compounds in AD model mice. The behavioral tests combined with the ultrastructure of TEM and IHC of different changes of Aβ-42 deposition in the profound lobe cortex indicate that the active compounds treatment was able to attenuate the cognitive and memory deficits associated with AD and substantiated the presence of neurotoxicity in the AD model mice. This may be associated with the inhibitory effects of Epimedium, Astragaoside, Puerarin on inflammatory, oxidative stress and apoptosis [15, 16, 19, 22]; 3) The iron gene and protein expression levels of four iron uptake protein, three iron release protein, one iron storage protein and iron regulated hormone were significantly altered in AD mice, and administration of Epimedium, Astragaoside, Puerarin active compounds had a beneficial effect on the changes in these iron metabolism related proteins; 4) The treatment of active compounds leads to an increased hepcidin level in the AD model mice, which may suppress accumulation of iron levels in the brain. Taken together, the compounds of effective components of Epimedium, Astragaoside, Puerarin improved memory and cognition impairment, decreased the accumulation of Aβ-42 in AD model mice, downregulated the expression of iron uptake protein DMT1-with/without IRE, TF, TfR1expression. Additionally, these compounds upregulated iron release protein FPN1, CP, HEPH expression and downregulated iron storage protein, Ferritin and iron regulated hormone, hepcidin. Hepcidin may have inhibited iron overload in the cerebral cortex of AD model mice, and at the same time relieved central nervous system protective functions under condition of iron overload. Thus, this study provides a new strategy for developing novel medications to treat AD.

Currently, one case of Alzheimer’s disease is occurring every 66 seconds. By the year of 2050, new cases of Alzheimer’s disease will be doubled as of today, which results in nearly 1 million new cases per year [23]. AD is the most common neurodegenerative disorder, clinically characterized by progressive memory loss and cognitive impairments. Although progress has been made in the past 20 years to understand AD pathophysiology [24], effective therapeutic treatments remain to be discovered. Therefore, the development of novel treatments for the AD is an urgent requirement in view of the rapidly grown populations with aging [23]. Iron chelating agents, such as DFO, has been shown beneficial effects on the AD, but it has a number of problems including side effects when it is taken orally. In contrast, the active compounds of Epimedium, Astragaoside and Puerarin may circumvent the shortcomings of chemical iron-chelators. Traditional Chinese medicine is a complete medical system with its own advantages. More specifically, the treatments were administered with Epimedium, Astragaoside and Puerarin, which are composed of active compounds, icariin, astragalosideIV and puerarin, respectively. Many studies have confirmed the uses of these active compounds in the treatment of neurological disorders [13, 18, 25]. Several studies have reported that these compounds may antagonize Aβ formation, scavenge free radicals, reduce inflammation, regulate mitochondrial functions and promote axonal maturation in the central nervous system [13, 18, 26, 27]. Icariin, a component extracted from Epimedium, has a beneficiary action on experimental spinal cord injury through its anti-oxidant, anti-neuroinflammatory, and anti-apoptotic role [28]. Our results showed that the active compounds could reduce the deposition of Aβ in the AD model mouse and improve the cognition including spatial and episodic memory. These data lay a theoretical foundation for the treatment of the AD using traditional Chinese medicine or a combination with Western medicine.

The toxicity of these Chinese medicines is one of the critical factors that need to be considered. For instance, it has been shown that prolonged usage of excessive amounts of Epimedium decreases thyroid activity and T3 while increases rT3 and TRH [29]. Also, puerarin may affect mouse embryonic development and viability [30]. Astragalus has a wide range of safety dosage and no toxicity associated with its usage has been reported [31]. However, limited data are available about the bioavailability of these herb extracts possibly due to that facts that is not clear about their effective components. Despite of these limitations, clinical studies have been performed to reveal their performance in human beings. Epimedium has beneficiary effect on patients with atherosclerosis, chronic obstructive pulmonary disease (COPD), diabetic nephropathy, polycystic ovarian syndrome (PCOS), and et al [32]. Puerarin possesses beneficial activities on cardiovascular diseases, neurological dysfunction, fever, and liver injury [33]. Astragalus has been widely used for immunomodulation, anti-oxidative and anti-inflammatory actions, and anti-cancer action [34].

The dosages used in this study are based on previous studies. Administration of icariin (30, 60, 120 mg/kg/day) for 17 consecutive days increased spatial learning and memory and reduced tumor necrosis factor-α (TNF-α), interleukin-1(IL1) and cyclooxygenase-2 (COX-2) expression in a rat model with brain inflammation induced by lipopolysaccharide (LPS) [35]. Puerarin’s low solubility and permeability result in poor gastrointestinal absorption and low bioavailability. Simply increasing the dose does not effectively increase bioavailability and might lead to toxicity and side effect [36]. Furthermore, Puerarin treatment markedly resulted in down-regulation of interleukin-6 (IL-6), tumor necrosis factor-α (TNF-α) and reactive oxygen species (ROS) with treated in Puerarin 80 mg/kg/day ig [37]. However, data about the pharmacokinetics and pharmacodynamics is limited because of many components are in these herb medicine. Future studies are warrantied to provide such information.

The four major iron uptake proteins are located in the cell membrane and are involved in the uptake and transport of iron ions. One of these proteins is DMT1 and according to the 3’-untranslational region of DMT1 mRNA with or without iron-response elements (IRE), it is also known as natural resistance-associated macrophage protein 2 (NRAMP 2). The DMT1 encoding gene, SLC11A2, is located on the long arm of chromosome 12 (12q13) and it is close to the susceptibility regions for AD and restless legs syndrome. Transferrin bind iron tightly, but reversibly. Transferrin has a molecular weight of around 77 kDa and contains two specific high-affinity Fe^3+^ binding sites. It has been reported that the iron saturation of transferrin is higher in AD and Down syndrome groups when compared with the controls, but the mechanism of transferrin in AD is unclear. Transferrin receptor protein 1 (TfR1), also known as Cluster of differentiation 71(CD71) is required for iron import from transferrin into cells by endocytosis. There is no research on the involvement of TfR1 in the mechanism of AD. A study showed that the administration of the active compounds of Epimedium, Astragaoside, Puerarin could improve the level of FPN1in the APP/PS1 transgenic mice model [38]. The structure of the ferritins are composed of 24 subunits including heavy (H) and light (L) chains, and they can deposit 4500 iron atoms as the storage iron protein in the cells. Previous studies have argued that high ferritin levels may contribute to an accelerated pathology in AD through involvement in the oxidative stress, which would promote the neuronal death [39, 40]. Additionally, ferritin levels have been suggested as an early diagnosis hallmark of AD. Our study results showed that the group of administration of active compounds Epimedium, Astragaoside and Puerarin could reduce the expression of these iron uptake and storage proteins and further represent the mechanism of the regulation function of hepcidin.

Several studies have indicated that the CP in the AD patients may be used as the new hallmarks in the serum [41]. Another ferric oxidase protein, HEPH, presents homology with CP, both of which participates in iron metabolism. The function of CP and HEPH converts iron (II) state, Fe^2+^, to iron (III) state, Fe^3+^, and mediates iron efflux most likely in cooperation with the iron transporter, FPN1. Previous study has pointed out that CP and HEPH are critical for CNS iron homeostasis and that a loss of CP and HEPH in the mouse leads to age dependent retinal neurodegeneration [42]. Recent studies have indicated that a deficiency in CP contributes to Parkinson’s disease through increased activities of iron accumulation and oxidative stress in the substantia nigra. A previous study has revealed that CP alterations in iron contents were mediated through DMT1-with IRE and changes in the ROS levels, which through the Erk/p38 and Bcl-2/Bax signaling pathways in turn attenuated the pathogenesis of AD [43]. CP through oxidation-induced structural changes enhanced the integrin-binding function may occur AD [44]. Our study results showed that the group administration of active compounds, Epimedium, Astragaoside and Puerarin could improve the expression of these iron release proteins compared to the AD model group. This likely occurs under the mechanism of the functional regulation of hepcidin. Hepcidin is a key regulator hormone of the iron entry in the circulation of the mammals, and it is secreted by the liver and controlled by iron stores within inflammation, macrophages, hypoxia, and erythropoiesis [45]. Hepcidin inhibits iron transport by binding to the iron export channel FPN1. The recent study has demonstrated that hepcidin could significantly reduce brain iron in the iron overloaded rats and suppressed transport of transferrin-bound iron from the periphery into the brain [46]. Our results showed that hepcidin could regulate the expression of the iron metabolism relative proteins in the cortex of AD model mouse.

**Figure 7:**
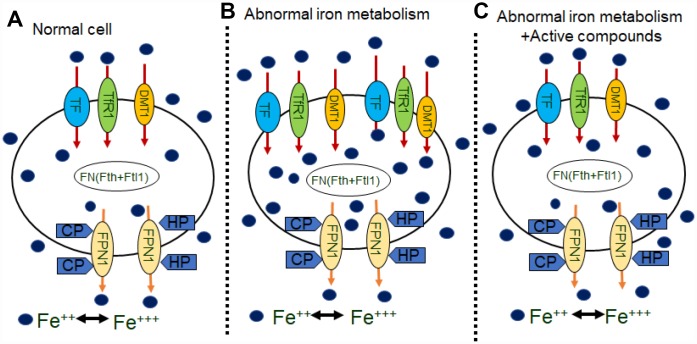
**Schematics illustrating the effects of active compounds on iron metabolisms in neurons.** (**A**) in normal cells, iron are transported into cells by TF, TfR1, or the DMT1 with/without IRE on the membrane. Some iron bind to FN (Fth or Ftl1) and then are transported out of cells by FPN1, HP, and CP. (**B**) In abnormal iron metabolism cells, increases in TF, TFR and DMT1 with/without IRE, which transport more iron into cells, and decreases in CP and HP, which transport less iron out of the cells, results an increase in intracellular iron concentration. (**C**) Application of active compounds reduces TF, TFR and DMT1 with/without IRE and increase CP, HP and FPN1 to recover intracellular iron concentration.

Alzheimer’s disease is a severe, progressive brain disorder that involved several mechanisms, including amyloid deposition theory, tau- protein phosphate theory, oxidative stress theory, genetic mutation theory and mitochondrial dysfunction theory. The study of iron in neurodegenerative disorders emerged a century ago, and abundant deposition of iron in the human brain have been observed in health and disease [47, 48]. Iron is an essential nutrient involved in many vital processes for life. Moreover, iron homeostasis is a strictly regulated process in healthy individuals through hydroxyl radical formation, protein aggregation, glutathione consumption, lipid peroxidation and nucleic acid modification [49]. Metal irons are implicated in the pathogenesis of AD [50, 51]. Therefore, altered iron homeostasis may be an important factor that leads to the pathogenesis of AD [52, 53] but the reasons for increased levels of iron in the brain of AD patients or animal model remain unclear. The iron metabolism related proteins are maintained in a balance to ensure that the iron content in the body can fulfill physiological needs. In this study we assume that the brain iron load may be associated with certain brain iron metabolism related proteins. We also assumed that the administration of the Chinese herb medicine of the compounds of Epimedium, Astragaoside and Puerarin could impact the uptake, release and storage of iron metabolism relative proteins in the brain tissues, which may be regulated by the ferric hormone, hepcidin. In above, the compounds of effective components of Epimedium, Astragaoside and Puerarin improved memory and cognitive decline, decreased the Aβ-42 in AD model mice, downregulated iron uptake protein DMT1-with/without IRE, TF, TfR1 expression, upregulated iron release protein FPN1, CP, HEPH expression and downregulated the iron storage protein Ferrtin and iron regulated hormone hepcidin. As a result, the effective components of Epimedium, Astragaoside and Puerarin inhibited iron overload in the cerebral cortex of AD model mice, relieved central nervous system protective functions in the condition of iron overload (Figure 7). Overall, this study provides a new strategy for developing novel medicines for treating the AD.

## MATERIALS AND METHODS

### Animals and treatments:

The present study was approved by the Ethics Committee of Hebei Medical University (Shijiazhuang, China). In total, 72 six-month-old male APP/PS1double transgenic mice and 12 male C57 mice (both obtained from Beijing HFK Bioscience Co., Ltd., Beijing, China) were used in this study. The APP/PS1 double transgenic mice were genotyped by polymerase chain reaction (PCR). The mice were housed under 12-h light/dark cycle conditions and were fed and watered regularly. The APP/PS1 double transgenic mice were randomly divided into six groups (12 mice/group), including treatment with 1) water, 2) the active components of Epimedium (120 mg/kg, intragastric administration, IG), 3) the active components of Astragaoside (80 mg/kg, IG), 4) the active components of Puerarin (80mg/kg, IG), 5) the mixture of three active components (IG), and 6) DFO (30 mg/kg, intramuscular injection, positive control). In addition, 12 male C57 mice also received water as the AD model control.

### Morris water maze

Behavioral examinations were conducted in a Morris water maze after three months of drug treatment, as described in the previous study. Briefly, a circular black pool (diameter, 120cm; depth, 55cm) was filled with water to a depth of 30 cm at 22°C. A clear circular platform (diameter, 10cm) was submerged 2 cm underwater in the northeast quadrant of the pool. Each mouse underwent four trials per day for six consecutive days. During the place navigation trial, mice were placed randomly into the pool facing the wall individually from four preset starting points, and were allowed to swim for a maximum of 90s or until they located the platform. On the sixth day, the spatial probe trial was conducted, in which the platform was removed from the pool and the mice were allowed to swim for 90s. The total swim time (escape latency); the number of times the animal crossed the previous location of the platform (platform-crossing); the time that the animal spent in the target quadrant where the platform is in; and the time of platform crossing and representative swimming paths were recorded using a video tracking system (SLYWMS Morris Water Maze System; Beijing Sunny Instruments Co., Ltd., Beijing, China).

### Novel object recognition

The novel object recognition test was performed to measure episodic memory. On day 1, mice were habituated to the open field area (40 × 40 × 30 cm) for 3 min. Twenty-four hours later on day 2, the time spent on investigating two identical objects (plastic toys) within the 5 cm distance in the open field as familiarity stage was recorded for 10 min with the ANYMaze video tracking program (Stoelting.co). Mice were then taken out of the area and returned to their home cages for 4 h. After 4 h one of the objects was replaced with a novel object of different shape and size and animals were then reintroduced into the area and allowed to explore the objects for 3 min in the exploratory stage. Time periods spent in the two stages were recorded. The preference of one object over another was assessed through the discrimination indices (DI) which is the time spent on the novel object (TN) relative to the time spent on both novel and familiar objects(TF): [DI = TN/(TN + TF)].

### Tissue preparation

Following the behavioral experiments, the mice were anesthetized with 50mg/kg sodium pentobarbital administered intraperitoneally, then euthanized by decapitation. Six brains were removed rapidly and divided into hemispheres on an ice-cooled board. The left and right cerebral cortex were dissected from each hemisphere and stored at 80°C for western blot and Real-time PCR analysis respectively. Three hemispheres were fixed in 4% paraformaldehyde (Tianjin Fu Chen Chemical Reagents Factory) and then in phosphate-buffered saline at 4°C overnight. The hemisphere was then embedded in paraffin (Tianjin Fu Chen Chemical Reagents Factory), cut into 4μm sections and stored at room temperature until required for Immunohistochemistry analysis.

### Transmission electron microscope

The cortex were separated and trimmed to obtain pieces of approximately 1.0 mm^3^ and were stored in 4% glutaraldehyde. And then perfused transcardially with 0.01M PBS containing heparin sodium for 2 minutes, followed by a 30-minute perfusion with 2.0% paraformaldehyde, 2.0% glutaraldehyde and 4% sucrose in 0.1M PBS. After perfusion, the animals were placed in sealed in plastic bags and kept in a refrigerator for 2 hours at 4° C. The brains were then removed and post-fixed in the same fixative at 4°C for at least 24 hours. Sectioning was performed in the coronal plane using a vibratome to obtain 200 μm thick sections. The sections were treated with 2% OsO4 in 0.1M PBS for 90 min at 4° C and rinsed again in 0.1 M PBS at room temperature. The sections were then dehydrated in a graded series of ethanol solutions, followed by propylene oxide, and left overnight in a mixture (1:1) of propylene oxide-Polybed 812 (Electron Microscopy Sciences, Hatfield, PA). Finally, the sections were flatly embedded in Polybed 812 in an oven at 60° C for 48–72 hours. The selection was made to include representative areas of unilateral groups.

### The mRNA expression in cerebral cortex as measured by quantitative real-time PCR

The left cerebral cortex was obtained from each mouse. Total RNA was extracted with Trizol. Optical density was measured at 260 and 280 nm using an ultraviolet spectrophotometer (Beckman Coulter, Kraemer Boulevard Brea, CA, USA). The purity and concentration of total RNA were calculated using the M-MLV First-Strand Synthesis System (Thermo, Prom-Test LLC, Koghbatsi 28/69, 0014, Yerevan, Armenia), 3μg of total RNA was added to a mixture of 1μL oligo(dT) [38] and RNase-free water was added up to 12μL. Samples were degenerated at 65°C for 5 minutes, and cooled on ice immediately. 4μL 5× reaction buffer, 1μL RiboLock RNase Inhibitor (20/ul), 2μL 10mM dNTP, 1μl RevertAid M-MuLV RT, total to 20μL, centrifuged and stirred briefly, The reaction product was stored at −20°C as cDNA samples. Primers were designed and synthesized by Invitrogen in accordance with each gene sequences in GenBank. Using the SYBR Green qPCRSuperMix-UDG with ROX (INVITROGEN) real-time PCR Kit. Standard curves of the target gene and β-actin gene amplification were established and their amplification efficiency was identified. A 25-μL volume reaction system consisted of 12.5 μL SYBR Ex Taq, 0.5 μL upstream primer (10 μM), 0.5 μL downstream primer (10 μM), 0.5 μL cDNA (10 μM) and 11 μL ddH2O. Amplification conditions consist of initial denaturation at 95°C for 3 minutes, denaturation at 95°C for 30 seconds, annealing for 30 seconds, and elongation at 72°C for 30 seconds for 40 cycles. Fluorescence was measured after each cycle. To identify the specificity of amplification, melting curves were analyzed after cycling. Experiments in each sample were performed in quadruplicate. The relative copy number of each sample was determined using BioRad Manager™ software (Bio-Rad Laboratories, Philadelphia, Armenia). Relative expression levels were calculated using the 2^–ΔΔCt^ method.

### Western blot analysis

1% Nonidet P40, 0.25% sodium deoxycholate, 1mM phenylmethanesulfonylfluoride, 10mg/ml leupeptin, 1 mM Na3VO4, 0.1% SDS and 1mM NaF. The lysates were collected, centrifuged at 80,000g for 20min and total proteins were quantified using a bicinchoninic acid kit (ZOMANBIO Biotech Co., Ltd., Hangzhou, China). The supernatant was removed after quantitative analysis denaturation then stored at 80°C. 30 μg of each total protein sample was subjected to SDS-PAGE using 8%, 10% and 15% gradient Tris/glycine gels and the separated proteins were transferred onto polyvinylidene difluoride membranes (EMD Millipore, Temecula, CA, USA). Subsequent to blocking in 5% nonfat milk for 2h, the membranes were incubated overnight with the following primary antibodies at 4°C: DMT1 with IRE (#NRAMP21-A;1:3,000) and DMT1 without IRE (#NRAMP23-A; 1:2,000) from Alpha Diagnostics International; Transferrin antibody (N3C3; 1:500) from GeneTex; TfR1 (1:200) from Thermo; FPN1 (1:3,000; #MTP11-A) from Alpha Diagnostics International, Ceruloplasmin (1:1000) from Invitrogen; Hephaestin (1:1000) from Alpha Diagnostics International; Ferritin (1:1000) from Abcam; and monoclonal rabbit anti-rat β-actin antibody (1:1,000) from sigma. The membranes were washed, then incubated with horseradish peroxidase conjugated goat anti-rabbit or anti-mouse IgG secondary antibody (1:10,000) for 2h. Protein levels were quantified from western blot analyses using Odyssey, and normalized against β-actin. The protein relative levels were calculated using the absolute gray value of each group divided by the C57 control group.

### Immunohistochemistry (IHC)

The paraffin sections were deparaffinized in xylene, rehydrated in distilled water, treated with 3% H_2_O_2_ for 30 min at 37°C to block endogenous peroxidase activity and washed with 0.1M PBS 3 times. Nonspecific binding was blocked by 10% goat serum for 40 min at 37°C and was followed by incubation overnight at 4°C with antibodies against the following: DMT1 with IRE (#NRAMP21-A; 1:2,000) and DMT1 without IRE (#NRAMP23-A; 1:2,000) from Alpha Diagnostics International; Transferrin antibody (N3C3; 1:250) from GeneTex;TfR1 (1:100) from Thermo; FPN1 (1: 2,000; #MTP11-A) from Alpha Diagnostics International, Ceruloplasmin (1:50) from Origene; Hephaestin (1:200) from Alpha Diagnostics International; Ferritin(1:200) from Abcam; Hepcidin (1:50) from Abcam. On the following day, sections were washed in 0.1M PBS and incubated with biotinylated goat anti-rabbit IgG for 25 min. Finally, the sections were washed in 0.1M PBS, stained with DAB, dehydrated using graded ethanol, and cleared using xylene. Images of positive staining in the frontal cortex region were captured at 20× magnification by a Nikon 80i microscope and analyzed using ipwin32 software to measure Integral optical density (IOD).

### Statistical analysis

Results were expressed as mean ± SEM. One-way analysis of variance followed by the Dunnet test was used to compare mRNA and IOD levels between different groups and MWM was used the repeated measures method. A probability value of p<0.05 was considered statistically significant.

### Ethics approval

All animal protocols were approved and conducted according to the recommendations from the Research Sub-Committee of Hebei Medical University on Animal Care and Use and the Chinese Council on Animal Care.
